# Pathological complete response of plasmacytoid variant bladder cancer to pembrolizumab following genomic analysis

**DOI:** 10.1002/iju5.12463

**Published:** 2022-05-09

**Authors:** Yusuke Goto, Satoki Tanaka, Masafumi Maruo, Sho Sugawara, Kazuto Chiba, Kanetaka Miyazaki, Atsushi Inoue, Tomohiko Ichikawa, Maki Nagata

**Affiliations:** ^1^ Yokohama Rosai Hospital Yokohama Kanagawa Japan; ^2^ Department of Urology Chiba University Graduate School of Medicine Chiba Japan

**Keywords:** bladder cancer, high tumor mutation burden, pembrolizumab, plasmacytoid variant

## Abstract

**Introduction:**

Plasmacytoid variant bladder cancer is a rare variant of urothelial carcinoma that accounts for 1% of bladder cancers. Plasmacytoid variant urothelial carcinoma is characterized by an aggressive phenotype and poor clinical outcomes.

**Case presentation:**

A 61‐year‐old woman presented with gross hematuria. Cystoscopy showed a 16‐mm solid tumor. Transurethral resection of the bladder tumor was performed, and the pathological diagnosis was invasive plasmacytoid variant urothelial carcinoma. Although the pathological T stage was pT1, computed tomography showed right obturator lymph node swelling. Since previous reports indicate poor response to chemotherapy for this disease, clinical sequencing was performed. Based on the high tumor mutation burden revealed, pembrolizumab was administered for 4 cycles, and computed tomography showed a partial response. Robot‐assisted radical cystectomy was performed, and a pathological complete response including the pelvic lymph node was observed.

**Conclusion:**

Pembrolizumab may be a treatment option for plasmacytoid variant urothelial carcinoma following genomic analysis.

**Abbreviations & Acronyms**
5‐ALA: 5‐aminolevulinic acid
CSI: carcinoma *in situ*
CT: computed tomography
DWI: diffusion‐weighted imaging
ICI: immune checkpoint inhibitor
MRI: magnetic resonance imaging
T2WI: T2‐weighted imaging
UC: urothelial carcinoma

**Keynote message**

Plasmacytoid variant bladder cancer is a rare variant with aggressive clinical features and poor outcomes. Immune checkpoint blockade following clinical sequencing could be a treatment option for this disease.

## Introduction

Plasmacytoid variant UC is a rare variant of UC that accounts for 1% of all UC cases, and it generally has a higher risk for progression to muscle‐invasive disease and locally advanced disease.^1^ The pathological diagnosis of plasmacytoid variant UC is reported to be associated with a locally advanced stage, a higher rate of positive surgical margins, and a higher rate of positive lymph node involvement compared with pure UC.^2^ The response rate to chemotherapy is relatively low, and novel approaches for the treatment of this disease are urgently needed. We report one case of plasmacytoid variant bladder cancer with lymph node metastasis successfully treated with pembrolizumab and surgical resection following the results of clinical sequencing demonstrating a high tumor mutation burden.

## Case presentation

**Fig. 1:**
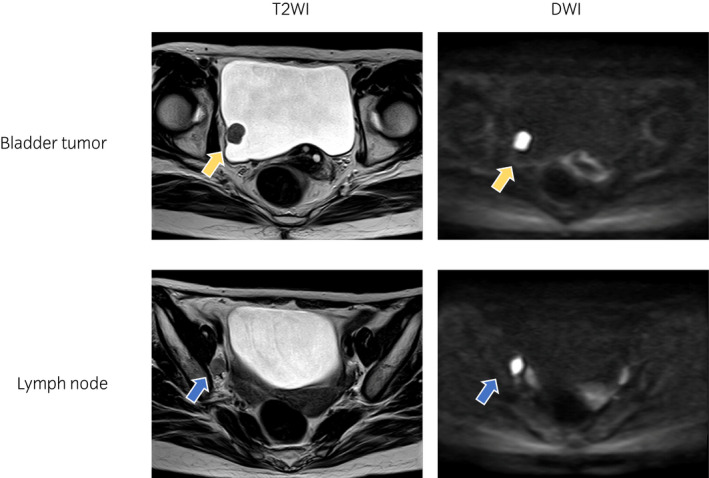
MRI of the bladder showed a solid tumor without muscle invasion (yellow arrow) and right obturator lymph node swelling (blue arrow). [Colour figure can be viewed at wileyonlinelibrary.com]

**Fig. 2:**
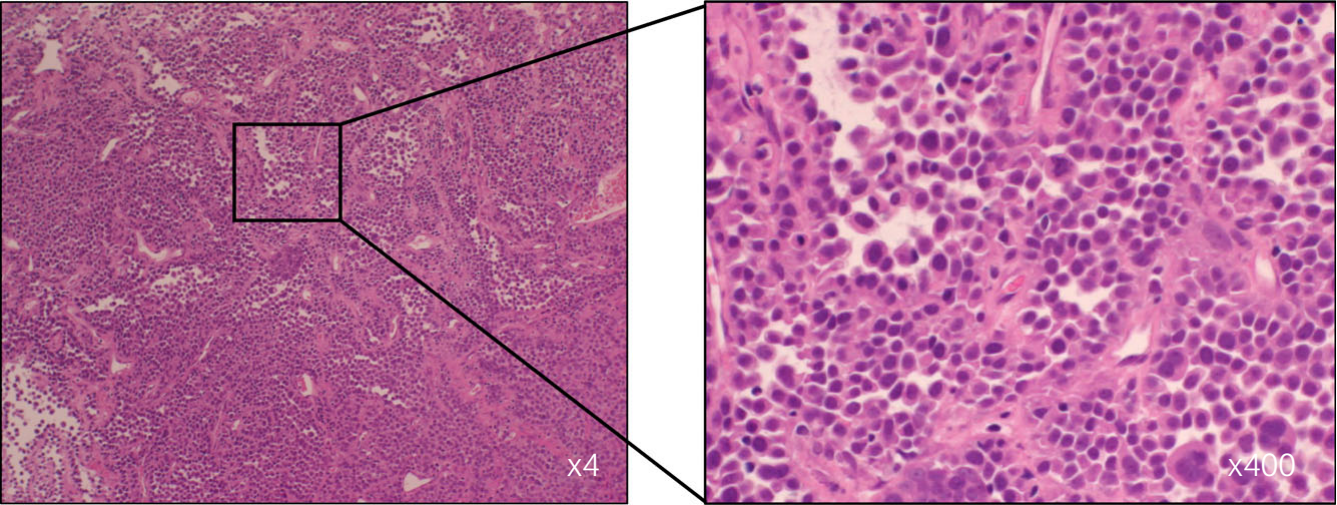
Hematoxylin and eosin staining of the bladder tumor. The pathological diagnosis was invasive plasmacytoid variant UC, high‐grade, pT1, with a CIS pattern. [Colour figure can be viewed at wileyonlinelibrary.com]

A 61‐year‐old woman presented to a local clinic with gross hematuria. Cystoscopy showed a 16‐mm solid tumor with a stalk on the right lateral wall of the bladder. Urine cytology was class IIIb. The patient was referred to our hospital for further treatment. MRI showed a solid tumor without muscle invasion and right obturator lymph node swelling (Fig. 1). Transurethral resection of the bladder tumor under general anesthesia showed histology positive for 5‐ALA. The pathological diagnosis was invasive plasmacytoid variant UC, high‐grade, pT1, with a CIS pattern (Fig. 2). Although the pathological T stage was pT1, MRI/CT showed right obturator lymph node swelling, but no other metastasis was identified, per se clinical N0M0. One cycle of chemotherapy with gemcitabine and cisplatin was administered; in the meantime, clinical sequencing was performed. Results of the Foundation One® CDx test showed a high tumor mutation burden with 21 mutations per 1 Mb, stable microsatellite status, a mutation in the *TERT* promoter, a deletion in *CDKN2A*, a deletion in *CDKN2B*, and a deletion in *CDH1*. Based on the high tumor mutation burden and progressive disease following one cycle of chemotherapy, the patient was treated with pembrolizumab for 4 cycles and achieved a partial response. She underwent robot‐assisted radical cystectomy with ileal conduit and lymph node dissection with pathology that revealed no residual carcinoma in the bladder and no positive lymph nodes (0 out of 34 lymph nodes). She has continued to receive pembrolizumab after surgery, with no evidence of recurrence or metastasis observed to date (1 year after cystectomy).

## Discussion

Plasmacytoid variant UC is a relatively rare and aggressive type of UC with high metastatic potential. Several retrospective studies have been reported. The largest was an analysis of 98 patients diagnosed with plasmacytoid variant UC.^2^ In that cohort, Li et al. showed that patients with plasmacytoid variant UC have a higher disease burden at radical cystectomy compared with those with pure UC. Furthermore, median overall survival for plasmacytoid variant UC was reported as 3.8 years, shorter than that of pure UC, which was reported as 8 years.^2^ Some case reports have shown a significant role for chemotherapy with cisplatin, but a retrospective cohort study of 81 patients with plasmacytoid variant UC and nonmetastatic disease showed only 21% downstaging with neoadjuvant chemotherapy, whereas 45% downstaging was obtained for patients with UC NOS, meaning that this disease has poor chemosensitivity.^3^, ^4^ Consequently, novel therapeutic options for this disease are warranted.

We conducted genomic analysis with Foundation One® CDx and obtained results indicating high tumor mutation burden; thus, we chose to administer pembrolizumab. In this case, the mutations included a mutation in the *TERT* promoter, a deletion in *CDKN2A*, a deletion in *CDKN2B*, and a deletion in *CDH1*. Similarly, recent reports showed a *CDH1* deletion in 20/33 (61%) cases and relatively higher tumor mutation burden compared with UC NOS.^4^ *CDH1* is *cadherin 1*, which encodes the E‐cadherin protein. The deletion in *CDH1* leads to loss of E‐cadherin expression, resulting in increased migratory function of the cancer cells, which is consistent with the aggressive clinical presentation of this disease. *CDKN2A* is one of the seven key bladder cancer genes (*FGFR3, CDKN2A, PPARG, ERBB2, E2F3, TP53*, and *RB1*) which are frequently altered in bladder cancer.^5^ Furthermore, a recent tumor microenvironment analysis showed that plasmacytoid UC was characterized by a luminal subtype, which may be sensitive to ICIs.^6^ Since we do not have access to the whole transcriptome data, we were not able to classify this case into consensus of the molecular classification of the muscle invasive bladder cancer, but the molecular classification of the plasmacytoid tumor could be helpful for understanding the molecular mechanisms. Although the biology of plasmacytoid UC is still unclear, these results indicate that plasmacytoid UC could be sensitive to ICIs, and use of ICIs may be a prudent treatment strategy after genomic analysis. Several previous papers showed efficacy to ICIs for plasmacytoid variant UC, but one report showed no response (Table 1).^4^, ^7^, ^8^ Large‐scale studies are needed to conclude ICI sensitivity for this rare variant.

**Table 1:** Previous reports of plasmacytoid variant UC treated with ICIs

No. of cases	Age	Gender	ICIs	Treatment line	Response	Reference no.
1	75	M	Pembrolizumab	Second line	PD: Died 4 months after TURBT	7
1	71	M	Pembrolizumab	First line	PR at 3 months, SD at 6 months	8
21	66 (55–76)	M/F = 13/8	19 cases: anti‐PD1/PDL1 2 cases: anti‐PD1 + anti‐CTLA4	First 8, Second 12, Fourth 1	Median PFS: 4.5 months Median OS: 10.5 months Median duration of response: 17.0 months	4

PD, Progressive disease.

In this case, we continued administration of pembrolizumab even after we confirmed pathological CR. A feature of pembrolizumab is a durable response, and a recent 5‐year follow‐up analysis of phase 3 KEYNOTE‐045 trial revealed a median duration of response of more than 2 years for patients with locally advanced or metastatic UC that progressed during or after platinum‐based chemotherapy.^7^ Another possibility was to discontinue pembrolizumab treatment after confirmation of the pCR at cystectomy, but considering the aggressive nature of the disease and the benefit of continued activation of CD8^+^ T cells, we decided to continue treatment with pembrolizumab. The expression level of CD8 or PD‐L1 of the TUR‐BT specimen can be possible factor for sensitivity to the pembrolizumab, consequently, can be the reason to continue pembrolizumab after cystectomy. No previous study has shown a role for continued ICI treatment for plasmacytoid variant UC after pCR on survival, and future clinical trials are needed.

## Conclusion

Our case demonstrated a pathological complete response of plasmacytoid variant bladder cancer to pembrolizumab. Plasmacytoid variant UC is an aggressive variant, but administration of pembrolizumab following genomic analysis could be a treatment option for this disease.

## Author contributions

Satoki Tanaka: Data curation; visualization. Masafumi Maruo: Data curation; resources. Sho Sugawara: Formal analysis; methodology; project administration. Kazuto Chiba: Data curation; investigation. Kanetaka Miyazaki: Data curation; investigation. Atsushi Inoue: Investigation; supervision. Tomohiko Ichikawa: Supervision. Maki Nagata: Supervision.

## Conflict of interest

The authors declare no conflict of interest.

## Approval of the research protocol by an Institutional Reviewer Board

Not applicable.

## Informed consent

We obtained informed consent from the patient.

## Registry and the Registration No. of the study/trial

Not applicable.
